# Supplementary material for: Development and Validation of a Mucosal Antibody (IgA) Test to Identify Persistent Infection with Foot-and-Mouth Disease Virus
Source: Viruses. 2021 May 1;13(5):814. doi: 10.3390/v13050814 (PMC8147266; doi:10.3390/v13050814)
Supplement: Supplementary file 1 [file viruses-13-00814-s001.zip › viruses-1185215-supplementary.pdf]

Table S1: Principal outcomes of four O1 Manisa/ O UKG vaccine challenge experiments. Four O1 Manisa/O UKG vaccine challenge experiments were carried out in the bio-security containment facility; Pirbright Labrotary, U.K. Animals in each experiment were assigned with 2 letter identifiers (UV, UY, VD/VE, and VH).

**Animal Experiment**					**No. of Vaccinated carriers by VI+RT-PCR**	**No of unvaccinated carriers by VI+RT-PCR**
Animal I.D.	Vaccine Dose	No. of Vaccinates	No. of unvaccinated controls	Days post vaccine challenge		
UV	1X O1 Manisa	20	5	21	9	2
UY	10x O1 Manisa	20	5	21	3	0
VD/VE	10x O1 Manisa	20	5	10	11	1
VH	1x O1 Manisa	20	5	10	9	2
